# P-1329. Invasive Streptococcal Infections in People Experiencing Homelessness in Metro Atlanta — Fulton, DeKalb, and Cobb County, Georgia, 2010–2022

**DOI:** 10.1093/ofid/ofae631.1507

**Published:** 2025-01-29

**Authors:** Sarah E Scott, Emily Mosites, Samantha Sefton, Stepy Thomas, Carla P Bezold, Monica M Farley

**Affiliations:** Emory University School of Medicine, Atlanta, Georgia; Multnomah County Health Department, Portland, Oregon; Emory University, Brookhaven, Georgia; Emory University, Brookhaven, Georgia; MITRE Corporation, Atlanta, Georgia; Emory University School of Medicine, Division of Infectious Diseases, Atlanta, Georgia

## Abstract

**Background:**

People experiencing homelessness (PEH) are disproportionately affected by infectious diseases compared to people who are housed (non-PEH). In the western U.S., 9–25% of invasive pneumococcal disease (IPD) and group A *Streptococcus* (IGAS) cases are among PEH; disparities in IPD and IGAS by housing status are not well-documented in other regions.

**Figure:**
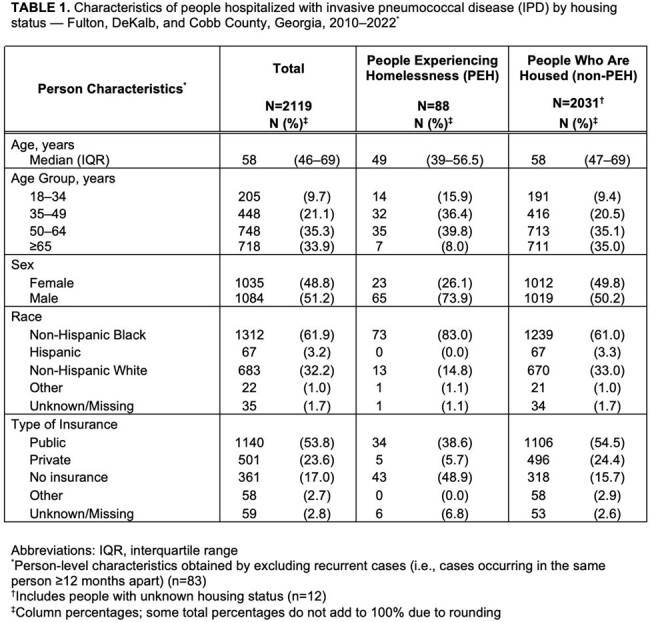

**Methods:**

Hospitalized IPD and IGAS cases among residents of 3 metro Atlanta counties (Fulton, DeKalb, & Cobb) ≥18 years occurring during 2010–2022 and their housing status at diagnosis were ascertained from the CDC-funded Georgia Emerging Infections Program. Recurrent cases ≤12 months apart were excluded. During 2010–2018, homelessness was defined by medical record documentation or residence in a shelter, mission, or medical respite. In 2019, the definition expanded to "unstable housing," which included transitional and single-room occupancy housing. Person-level case characteristics by housing status were calculated. Annual age-adjusted incidence per 100,000 population by housing status and incidence rate ratios with 95% confidence intervals were estimated using Poisson regression. Point-in-Time counts (PEH) and 2020 US Census data (non-PEH) were used as population denominators.

**Figure:**
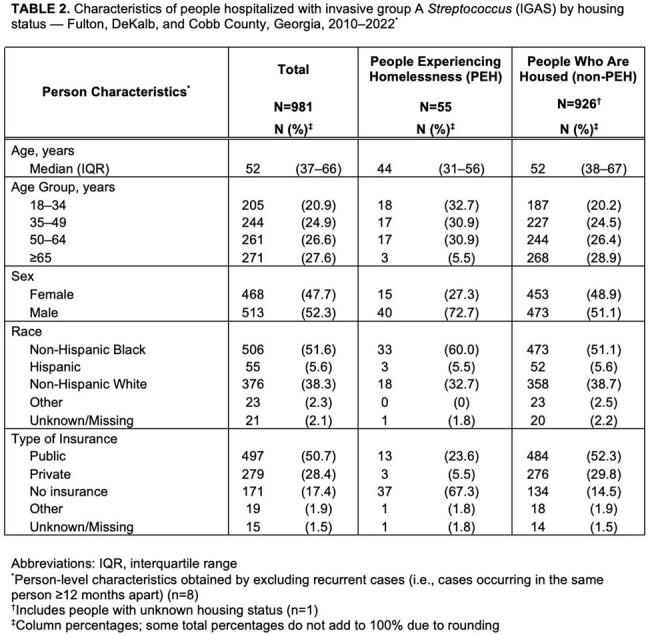

**Results:**

During 2010–2022, 2,202 IPD cases in 2,119 people and 989 IGAS cases in 981 people were identified. Among people with IPD and IGAS, 88 (4%) and 55 (6%) were PEH, respectively (Tables 1–2). IPD and IGAS incidence rates were higher among PEH than non-PEH (Table 3). Among PEH with IPD, 65 (74%) were men and 81 (92%) were < 65 years (vs. 1019 [50%] and 1320 [65%] in non-PEH). Among PEH with IGAS, 40 (73%) were men and 52 (95%) were < 65 years (vs. 473 [51%] and 658 [71%] in non-PEH). PEH with IPD or IGAS were more likely to lack insurance. Both IPD and IGAS incidence among PEH increased before and after the homeless definition change; among non-PEH, incidence decreased (IPD) or was stable (IGAS) (Fig 1).

**Figure:**
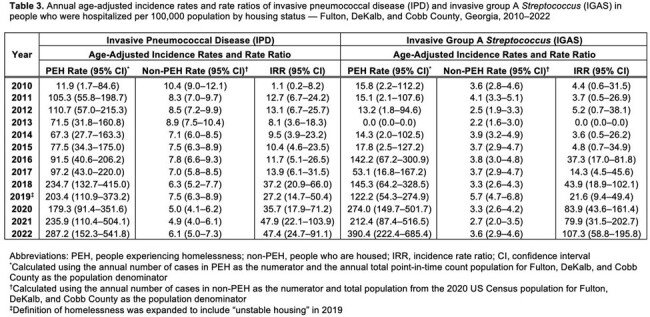

**Conclusion:**

The rate of IPD and GAS cases was higher among PEH than non-PEH and increased during 2010–2022. IPD and IGAS cases among PEH occurred more frequently in men and people < 65 years compared to non-PEH. Disparities in IPD and IGAS incidence by housing status exist within metro Atlanta and may be increasing. PEH should be prioritized in IPD and IGAS prevention efforts.

**Figure:**
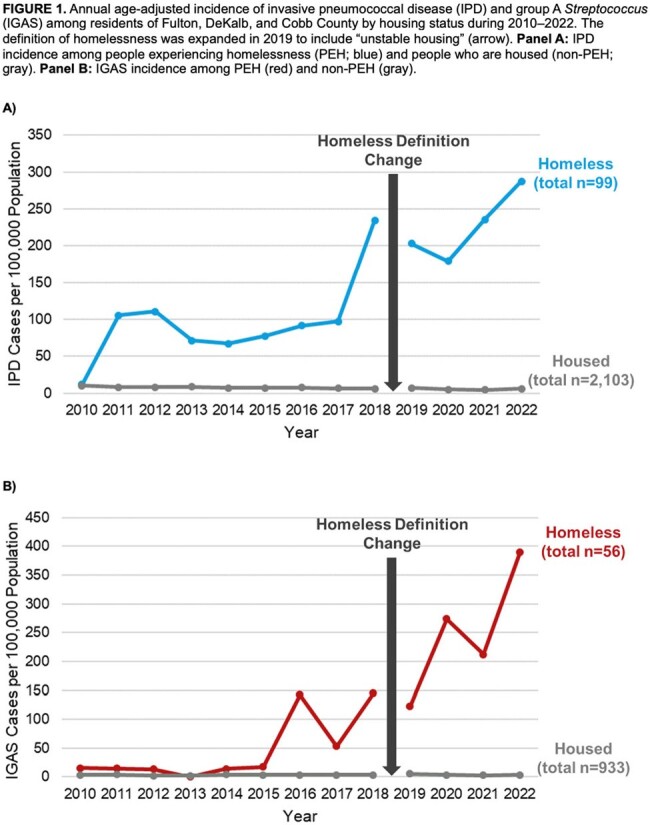

**Disclosures:**

**All Authors**: No reported disclosures

